# Kaposi Sarcoma in Mantled Guereza

**DOI:** 10.3201/eid2508.181804

**Published:** 2019-08

**Authors:** Anna Grewer, Martina Bleyer, Kerstin Mätz-Rensing, Alexander S. Hahn, Tim Rüggeberg, Gregor Babaryka, Andre Zimmermann, Stefan Pöhlmann, Artur Kaul

**Affiliations:** Zoo Krefeld GmbH, Krefeld, Germany (A. Grewer);; German Primate Center–Leibniz Institute for Primate Research, Göttingen, Germany (M. Bleyer, K. Mätz-Rensing, A.S. Hahn, S. Pöhlmann, A. Kaul);; Heinrich-Heine-University Düsseldorf, Düsseldorf, Germany (T. Rüggeberg, G. Babaryka, A. Zimmermann);; Joint Practice for Oral and Maxillofacial Surgery, Neuss, Germany (A. Zimmermann);; University of Göttingen, Göttingen (S. Pöhlmann)

**Keywords:** Kaposi sarcoma, Kaposi’s sarcoma herpesvirus, KSHV, nonhuman primates, rhadinovirus, viruses, mantled guereza, Colobus guereza kikuyensis, Colobine gammaherpesvirus 1, zoonoses

## Abstract

We identified a novel Kaposi’s sarcoma herpesvirus–related rhadinovirus (Colobine gammaherpesvirus 1) in a mantled guereza (*Colobus guereza kikuyensis*). The animal had multiple oral tumors characterized by proliferation of latent nuclear antigen 1–positive spindle cells and was not co-infected with immunosuppressive simian viruses, suggesting that it had Kaposi sarcoma caused by this novel rhadinovirus.

Kaposi’s sarcoma herpesvirus (KSHV), a member of the genus *Rhadinovirus*, is the causative agent of Kaposi sarcoma (*1*), an endothelial neoplasm of the dermis, oral cavity and intestinal organs. The tumors are highly vascularized and characterized by proliferation of spindle cells that contain KSHV DNA and antigen (*2*,*3*). Predisposing factors for Kaposi sarcoma include immunodeficiency, especially infection with HIV (*4*). Nevertheless, a major portion of Kaposi sarcoma cases in Africa occurs in HIV-negative persons (*5*).

Clinically, Kaposi sarcoma is divided into 4 forms: classical Kaposi sarcoma, African endemic Kaposi sarcoma, Kaposi sarcoma caused by iatrogenic immunosuppression, and HIV-associated Kaposi sarcoma (*6*,*7*). Lesions of classical Kaposi sarcoma initially occur on the lower extremities, progress slowly, and affect visceral organs at a late stage (*6*,*7*). In contrast, the remaining Kaposi sarcoma forms affect lymph nodes, mucosa, and visceral organs at early stages, progress rapidly, and encompass symptoms in the hard palate and oral mucosa (*6*,*7*).

Rhadinoviruses with high similarity to KSHV have been detected in Old World monkeys, including chimpanzees (*8*), macaques (*9*–*11*), and African green monkeys (*12*). The rhadinoviruses have split into 2 lineages, RV1 and RV2, and many Old World monkeys harbor viruses of both lineages. In contrast, humans harbor only KSHV, which belongs to the RV1 lineage. Kaposi sarcoma–like disease has been observed in rhadinovirus-infected nonhuman primates (NHP), but only in the presence of immunodeficiency, induced, for instance, by co-infection with simian immunodeficiency virus (*13*,*14*).

We report Kaposi sarcoma in a simian immunodeficiency virus– and simian retrovirus–negative mantled guereza (*Colobus guereza kikuyensis*) that was infected with a novel rhadinovirus that had high homology to KSHV. This new virus is called Colobine gammaherpesvirus 1 (CbGHV1).

## The Study

**Figure 1:**
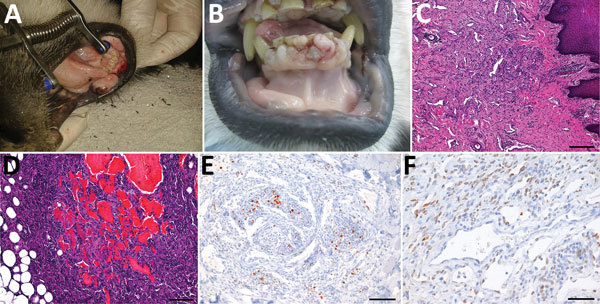
Disease manifestations in mantled guereza with Kaposi sarcoma. A) Oligofocal flattened masses on the inner aspects of the lower lip. B) Multinodular fissured masses at the gingival margin. C) Fibrovascular stroma in the subepithelial propria of the lower lip with spindle cell proliferations delineating narrow vascular clefts and containing lymphoplasmacytic inflammatory cell infiltrates, hematoxylin and eosin stained; scale bar indicates 200 µm. D) Spindle cell proliferation with cavern formation in the perinodal adipose tissue of the mandibular lymph node; hematoxylin and eosin stained; scale bar indicates 100 µm. E) Immunohistochemical staining showing variable Ki67 expression in <20% of spindle cells, streptavidin-biotin complex method–diaminobenzidine tetrahydrochloride; scale bar indicates 100 µm. F) Immunohistochemical staining showing nuclear expression of latent nuclear antigen 1 in ≈50%–60% of spindle cells, streptavidin-biotin complex method–diaminobenzidine tetrahydrochloride; scale bar indicates 50 µm.

A 13-year-old female mantled guereza who was born in a zoological garden in Germany showed development of swelling on the inner aspects of the lower lips; several circumscribed masses were found on the inner upper and lower labial mucosa. The masses were pink to light red and had a smooth and shiny surface, coarse consistence, and a diameter of 1–2 cm (Figure 1, panel A). After incision of 1 mass, the surface of the cut appeared to be cavernous and highly vascularized.

The mucosal masses were removed by surgery. Subsequently, bilateral cataract, progressive weight loss, and recurrence of the mucosal masses developed in the animal, and it had to be euthanized (Appendix). Necropsy showed several flattened and smooth tumorous lesions on the inner aspects of the upper and lower lips, as well as multiple small, partly ulcerated nodules at the gingival margin of the upper and lower jaw (Figure 1, panel B).

Histologically, masses and nodules consisted of a collagen-rich fibrous stroma with multifocal areas of increased cellularity represented by spindle cell proliferations with moderate lymphoplasmacellular infiltrates (Figure 1, panel C). The tonsils and mandibular and axillary lymph nodes showed similar foci of fibrovascular tissue. In the perinodal adipose tissue of 1 mandibular lymph node, we found distinct formation of caverns lined by endothelial cells, filled with erythrocytes, and surrounded by spindle cells (Figure 1, panel D).

**Figure 2:**
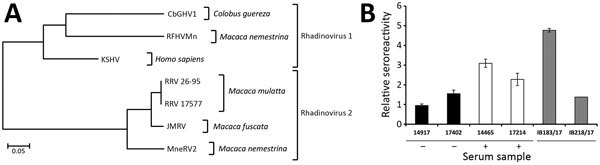
Analysis of CbGHV1 and seroreactivity in mantled guereza with Kaposi sarcoma. A) Phylogenetic analysis of partial sequences of the polymerase gene. Analysis was performed by using the neighbor-joining method. The distance between CbGHV1 and selected viruses was analyzed by using the maximum composite–likelihood method and MEGA6 (https://www.megasoftware.net). The PCR sequence of CbGHV1 was compared with KSHV (GenBank accession no. NC_009333.1); RFHVMn (KF703446.1); RRV 26–95 (AF210726.1); RRV 17577 (NC_003401.1); JMRV (AY528864.1); and MneRV2 (KP265674.2). Scale bar indicates nucleotide substitutions per site. B) Antibodies from mantled guereza with Kaposi sarcoma showing cross-reactivity against KSHV. Reactivities of KSHV antibody–positive human serum samples (14465 and 17214), KSHV antibody–negative human serum samples (14917 and 17402), and serum sample from the Kaposi sarcoma–affected mantled guereza (IB183/17) and its healthy offspring (IB218/17) were analyzed by ELISA. Relative reactivities of serum samples with KSHV-positive and KSHV-negative cell lysates are shown. The sum of relative errors is used as an error estimate for the ratio and is indicated by error bars (mean ± half error). Reactivity of human serum samples against KSHV is indicated. CbGHV1, Colobine gammaherpesvirus 1; JMRV, Japanese macaque rhadinovirus; KSHV, Kaposi’s sarcoma herpesvirus; MneRV2, *Macaca* *nemestrina* rhadinovirus 2; RFHVMn, retroperitoneal fibromatosis–associated herpesvirus *M. nemestrina*; RRV, rhesus rhadinovirus; –, negative; +, positive.

Immunohistochemical examination showed distinct immunoreaction of most spindle cells with endothelial cell markers CD31 and von Willebrand factor. We found variable expression of Ki67 in <20% of spindle cells (Figure 1, panel E), and ≈50%–60% of spindle cells reacted with antibodies against KSHV latent nuclear antigen (Figure 1, panel F). These findings were compatible with Kaposi sarcoma. A pan herpesvirus PCR amplified DNA fragments in all tested samples (blood, swabs, pathologic tissues of upper and lower lips) that contained identical sequences of a novel herpesvirus (CbGHV1). This virus is most closely related to retroperitoneal fibromatosis herpesvirus from *Macaca nemestrina* (the pig-tailed macaque) (Figure 2, panel A) and was also identified in a sibling of the animal we studied (*15*).

We found high viral loads in the tumorous masses of the oral cavity, in swabs from cut surfaces of mucosal masses, and in tumorous lesions on the inner upper and lower lips, as well as in nodules at the gingiva margin (Table 1). A lower viral load was detected in blood and was identical to that measured in the blood of a healthy offspring of the animal (Table 1). Viral load in all remaining organs was in the range of that measured for blood, potentially because of circulation of positive blood cells through these organs. Moreover, mucosal, anal and fluid swab samples were clearly positive for viral genomes and the high viral load in the mucosa of the eye and in the lacrimal glands might be explained by Kaposi sarcoma in unusual locations (*16*). Finally, CbGHV1-negative samples were not available for calibration by PCR; their inclusion might have altered overall, but not relative, CbGHV1 genome copies measured.

**Table 1:** Viral loads of CoRhV genomes in various organs of mantled guereza with Kaposi sarcoma*

Category	Copies of CbGHV1 DNA/μg total DNA ± SD
Sampling during anesthesia	
Blood	4 × 10^4^ ± 8 × 10^3^
Blood	7 × 10^4^ ± 2 × 10^4^†
Mucosal masses	
Swab specimen from cut surface	6 × 10^7^ ± 3 × 10^7^
Buccal	9 × 10^6^ ± 1 × 10^6^
Upper labial	3 × 10^7^ ± 6 × 10^6^
Lower labial	2 × 10^7^ ± 3 × 10^6^
Sampling during necropsy	
Mucosal masses	
Gingiva	9 × 10^5^ ± 1 × 10^5^
Upper labial	6 × 10^6^ ± 1 × 10^6^
Lower labial	1 × 10^7^ ± 5 × 10^6^
Oral swab specimen	5 × 10^3^ ± 2 × 10^3^‡
	5 × 10^4^ ± 2 × 10^4^‡
Genital swab specimen	6 × 10^4^ ± 1 × 10^4^
Anal swab specimen	3 × 10^5^ ± 5 × 10^4^
Lacrimal fluid swab specimen	3 × 10^6^ ± 6 × 10^5^
Spleen	5 × 10^5^ ± 9 × 10^4^
Kidney	6 × 10^3^ ± 3 × 10^3^‡
Kidney	2 × 10^5^ ± 2 × 10^4^‡
Liver	3 × 10^3^ ± 2 × 10^3^
Lung	1 × 10^4^ ± 6 × 10^3^
Heart	9 × 10^3^ ± 3 × 10^3^
Brain	1 × 10^4^ ± 5 × 10^3^

*Values are mean ± SD for 3 independent quantitative PCRs. CbGHV1, Colobine gammaherpesvirus 1. †Results for healthy offspring. ‡Two samples were obtained during necropsy.

Serologic analysis showed that the guereza had antibodies against lymphocryptovirus, cytomegalovirus, and simian foamy virus but, somewhat counter intuitively, not against rhesus rhadinovirus (RRV) (Table 2). In contrast, serum from the animal was reactive against KSHV antigen in an ELISA (Figure 2, panel B) and an immunofluorescence-based assay (data not shown). Finally, we did not detect antibodies reactive against KSHV in a CbGHV1-positive healthy offspring, potentially because CbGHV1 antibody levels were higher in diseased compared with healthy animals.

**Table 2:** Antibodies against selected viral antigens in mantled guereza with Kaposi sarcoma*

Antigen source	Test result	
	Monkey with Kaposi sarcoma	Healthy offspring
Herpes simplex viruses	–	–
Simian immunodeficiency virus	–	–
Simian retrovirus	–	–
Simian T-cell leukemia virus	–	–
Measles virus	–	–
Rhesus rhabdovirus	–	–
Lymphocryptovirus	+	+
Cytomegalovirus	+	+
Simian foamy virus	+	–

*–, negative; +, positive.

## Conclusions

The animal we studied had several characteristic features of Kaposi sarcoma, including tumorous lesions in the buccal mucosa and proliferation of spindle cells, which harbored viral antigen. Although the disease symptoms did not fully match those of Kaposi sarcoma in humans (*3*,*4*), in part because of absence of initial symptoms in the lower extremities, an animal model based on CbGHV1 might still provide major insights into Kaposi sarcoma/KSHV infection of humans.

CbGHV1 exhibited a higher similarity to KSHV and retroperitoneal fibromatosis herpesvirus from *M. nemestrina,* which are RV1 rhadinoviruses, when compared with RRV, a RV2 rhadinovirus. Consistent with these findings, serum from the guereza cross-reacted with KSHV but not RRV. However, we cannot exclude that assay specificity was moderate and confirmation with independent tests is pending. Apart from the animal having Kaposi sarcoma, 4 genetically related animals were also PCR-positive for CbGHV1, raising questions regarding the route of transmission. We detected high copy numbers of the viral genome in swab specimens of the oral cavity and the anogenital mucosa, suggesting that transmission might occur by close contact, including sex, the route of KSHV transmission between humans (*3*,*4*).

CbGHV1 was most likely involved in tumorigenesis because high numbers of the viral genome were found within tumorous tissues. Moreover, viral antigen was detected in spindle cells. However, it was unclear that infection by CbGHV1 was sufficient to induce Kaposi sarcoma. A link between immunosuppression and Kaposi sarcoma has been established for human patients and cannot be excluded for CbGHV1/NHP (*3*,*4*). A younger male sibling of the guereza analyzed in this study was PCR positive for CbGHV1 and showed development of primary effusion lymphoma, another disease caused by KSHV, without evidence for immunosuppression (*15*). Thus, a genetic component might contribute to disease development. Finally, 1 offspring of the animal infected with Kaposi sarcoma and 2 genetically related animals were also CbGHV1-positive but healthy; it remains to be examined whether they will show development of disease in the future.

We report a case of spontaneous Kaposi sarcoma in an NHP. Our findings might aid the development of an NHP model for KSHV/Kaposi sarcoma in humans. For development of this model, it is critical to isolate CbGHV1; those efforts are under way.

Appendix Additional information on Kaposi sarcoma in mantled guereza.
