# Supplementary material for: Kaposi Sarcoma in Mantled Guereza
Source: Emerg Infect Dis. 2019 Aug;25(8):1552–5. doi: 10.3201/eid2508.181804 (PMC6649314; doi:10.3201/eid2508.181804)
Supplement: Appendix — Additional information on Kaposi sarcoma in mantled guereza. [file 18-1804-Techapp-s1.pdf]

Article DOI: https://doi.org/10.3201/eid2508.181804

Kaposi Sarcoma in Mantled Guereza

Appendix

Methods

Anesthesia and Biopsies

General anesthesia was performed by using 0.7 mg/kg xylazine (2% Xylazin; Serumwerk Bernburg AG, http://www.serumwerk.com) and 10 mg/kg ketamine (Narketan [100 mg/mL]; Vétoquinol GmbH, https://www.vetoquinol.de) intramuscularly and was maintained with isoflurane (isoflurane CP [1 mL/mL]; CP-Pharma Handelsgesellschaft mbH, https://www.cp-pharma.de). Local vasoconstriction in the mucosa of the oral cavity was achieved by subcutaneous administration of ultracain plus adrenalin (Ultracain D-S [2 mL]; Sanofi-Aventis Deutschland GmbH, https://www.sanofi.deGmbH) at a ratio of 1:200,000.

Biopsy specimens from different tumorous masses were obtained and fixed in 10% buffered formaldehyde for routine histologic examination. In addition, serum samples, blood (in EDTA), and aerobic, anaerobic and native swab specimens from the cut surface of the masses, as well as tumorous tissue of the buccal area (upper lip and lower lip) were obtained for viral testing that included a pan herpesvirus nested PCR. Biopsy sites were closed intracutaneously by using a resorbable intracutaneous suture. The animal was treated with 0.2 mg/kg meloxicam (Metacam [5 mg/mL]; Boehringer Ingelheim Vetmedica GmbH, https://www.bi-vetmedica.com) and 10 mg/kg long-acting amoxicillin (Duphamox LA [150 mg/mL]; Zoetis Deutschland GmbH, https://www.zoetis.de) intramuscularly.

Anesthesia was partly antagonized by administration of atipamezol (Nosedorm [5 mg/mL]; Alfavet Tierarzneimittel GmbH, https://www.alfavet.de/startseite.html). After recovery from anesthesia, drug therapy was supplemented orally with 20 mg omeprazole, 1×/d (Antra Mups [20 mg]; AstraZeneca GmbH, https://www.astrazeneca.de), 0.2 mg/kg meloxicam (Metacam [1.5 mg/mL]; Boehringer Ingelheim Vetmedica GmbH), and 1 dose of Zylexis (Zoetis Deutschland GmbH) on days 0, 2, and 7 after surgery.

Immunohistochemical Analysis

Immunohistochemical analysis was performed on paraffin-embedded sections by using the following primary antibodies: Ki67 antibody (monoclonal mouse antihuman Ki67 antigen, clone MIB-1 [1:50 dilution]; DakoCytomation GmbH, https://www.agilent.com) CD31 antibody (monoclonal mouse antihuman endothelial cell antibody, clone JC70A [1:10 dilution]; DakoCytomation GmbH), von Willebrand factor antibody (monoclonal mouse antihuman von Willebrand factor antibody, clone F8/86 [1:25 dilution]; DakoCytomation GmbH), and antihuman herpesvirus 8 antibody (monoclonal rat antihuman herpesvirus 8 HHV8 antibody monoclonal, clone LN35 [1:10 dilution]; Abcam plc, https://www.abcamplc.com). Immunohistochemical staining was performed by using an automated immunostaining system (Discovery XT; Roche Diagnostics GmbH, Mannheim, Germany), the streptavidin-biotin complex method, diaminobenzidine tetrahydrochloride for signal detection (DAB Map Kit, Roche Diagnostics GmbH, https://www.roche.com), and appropriate positive and negative controls.

Extraction of Total DNA from Samples

Total DNA was isolated from the indicated tissue samples by using the First-DNA All-Tissue Kit (GEN-IAL GmbH, https://www.gen-ial.de) according to the manufacturer’s instructions. Concentration of extracted DNA was quantified by means of optical density (OD) measurements, and total DNA was adjusted to a concentration of 0.1 µg/µL. Subsequently, all DNA concentrations were verified by 3 OD measurements.

Qualitative PCR and Sequencing

Viral sequences were amplified by PCR from total DNA isolated from blood (in EDTA), a swab specimen obtained from cut surface of the masses, and tumorous tissues of the buccal area, upper lip, and lower lip. PCR amplification was performed by using previously reported pan erpesvirus PCR nested primer sets (*1*). The final volume of the PCR was 25 µL and contained the following components: 0.5 mmol/L $MgCl_2$, 2.5% (vol/vol) dimethylsulfoxide, 0.2 mmol/L deoxynucleoside triphosphates, 1.2 µmol/L of each primer, 1× PCR buffer, and 0.25 µmol/L HotStarTaq DNA Polymerase (QIAGEN, https://www.qiagen.com). For amplification of the target sequences, samples were first heated to 95°C for 4.5 min; 55 cycles were then run with 1 cycle consisting of melting (20 s at 95°C), annealing (30 s at 46°C or 20 s at 46°C for nested PCR amplification), and extension (30 s at 72°C or 20 s at 72°C for nested PCR amplification).

Samples were then incubated for 10 min at 72°C. PCR products were extracted from agarose gels after electrophoresis by using a commercial kit (NucleoSpin Gel and PCR Clean-Up; Macherey-Nagel, https://www.mn-net.com) according to the manufacturer's instructions and sequenced by using Sanger sequencing.

Quantitative PCR

For quantitative analysis of viral loads, primers and probes were designed that were specific for the polymerase gene. A GenScript (https://www.genscript.com) real-time PCR (TaqMan) primer design was used as a tool for design of the following oligonucleotides: sense primer: 5′-CCGAGACAGTAACCCTCCAA-3′, antisense primer: 5′-TTAGCAGGCAGGCTAAGTGT-3′, and probe of antisense polarity: 5′-FAM-TGGCTTCCACGAAGACCTGTGACT-BHQ-1-3′ (Microsynth, https://www.microsynth.ch). Two microliters of DNA extractions were used for quantitative PCR analysis by using Rotor-Gene Q (QIAGEN). Amplifications were conducted in duplicate and PCRs were repeated 3 times. For PCR, samples were first heated to 95°C for 15 min; 45 cycles were then run with each cycle consisting of melting (15 s at 95°C) annealing and extension (1 min at 60°C). The total volume of the reaction mixture was 25 μL and contained the following components: 3 mmol/L $MgCl_2$, 0.4 mmol/L deoxynucleoside triphosphates, 0.266 μmol/L probe, 0.6 μmol/L (each) sense and antisense primers, 1× PCR buffer, and 0.25 μL HotStarTaq DNA Polymerase (QIAGEN).

Chip-Based Technology for Detection of Antibodies

For simultaneous detection of serum antibodies to herpes simplex viruses, simian immunodeficiency virus, simian retrovirus of type D, simian T-cell leukemia virus, measles virus, rhesus rhadinovirus, lymphocryptovirus, cytomegalovirus, and simian foamy virus, we used a commercial microarray multiplexing technology (Simian Panel E Kit; Intuitive Biosciences, http://intuitivebio.com). All steps were performed according to the manufacturer's instructions. Subsequently, microarrays were scanned and analyzed by using the image capture and analysis system AQ 1000 (Intuitive Biosciences).

Preparation of Antigens and Kaposi's Sarcoma Herpesvirus ELISA

ELISA coating material was prepared by induction of either empty iSLK cells or iSLK cells harboring Kaposi's sarcoma herpesvirus BAC16 with 2.5 mmol/L sodium butyrate and 1 μg/mL doxycycline for 2 days. Cells were then harvested by brief trypsinization and collected by centrifugation. Pelleted cells and debris were lysed in phosphate-buffered saline (PBS) containing

1% NP40 for 1 h on ice. The lysate was clarified by centrifugation for 2 h at 4,200 rpm in a TX-1000 swing bucket rotor (ThermoFisher Scientific, https://www.thermofisher.com) in 50-mL tubes, adjusted to a concentration of 0.7 mg/mL, as measured by using the Bradford assay, aliquoted, and stored at −80°C. A total of 50 µL of this material per well was used for coating ELISA plates overnight at 4°C. Before use, plates were washed 3 times (5 min/wash) with 200 µL PBS containing 0.5% Tween 20 (PBS-T) per well. The wells were blocked with 300 µL blocking buffer (10% fetal calf serum in PBS-T) for 2 h at 37°C. Serum samples were diluted 1:40 in blocking buffer, and 50 µL were added per well in duplicates after removal of the blocking buffer, followed by incubation for 2 h at 37°C. After 3 washes with 200 µL PBS-T, 100 µL of goat antihuman IgG (heavy + light chain) horseradish peroxidase conjugate (Southern Biotech, https://www.southernbiotech.com) at a 1:12,000 dilution in blocking buffer was added, and plates were incubated for 1 h at 37°C. Plates were then washed 3 times with 200 µL PBS-T, 100 µL substrate solution (3,3′,5,5′ tetramethylbenzidine; Sigma-Aldrich, https://www.sigmaaldrich.com) was added, and incubated for 20 min at 37°C. After addition of 100 µL stop solution (1 mol/L HCl), OD was determined at 450 nm. All samples were measured in duplicate for each coating. The ratio of the ODs after background subtraction was calculated. The relative range of each duplicate was calculated and used as an error estimate. The sum of the 2 relative errors was used as an error estimate for the ratio and is represented by error bars (mean ± half error).

Reference

1. Chmielewicz B, Goltz M, Lahrmann KH, Ehlers B. Approaching virus safety in xenotransplantation: a search for unrecognized herpesviruses in pigs. Xenotransplantation. 2003;10:349–56. PubMed https://doi.org/10.1034/j.1399-3089.2003.02074.x
